# A Cost-Effective *Pichia pastoris* Cell-Free System Driven by Glycolytic Intermediates Enables the Production of Complex Eukaryotic Proteins

**DOI:** 10.3390/bioengineering11010092

**Published:** 2024-01-18

**Authors:** Jeffrey L. Schloßhauer, Srujan Kumar Dondapati, Stefan Kubick, Anne Zemella

**Affiliations:** 1 Fraunhofer Project Group PZ-Syn of the Fraunhofer Institute for Cell Therapy and Immunology (IZI), Branch Bioanalytics and Bioprocesses (IZI-BB), Located at the Institute of Biotechnology, Brandenburg University of Technology Cottbus-Senftenberg, 01968 Senftenberg, Germany; 2 Fraunhofer Institute for Cell Therapy and Immunology (IZI), Branch Bioanalytics and Bioprocesses (IZI-BB), Am Mühlenberg, 14476 Potsdam, Germanystefan.kubick@fu-berlin.de (S.K.); 3 Laboratory of Protein Biochemistry, Institute for Chemistry and Biochemistry, Freie Universität Berlin, Thielallee 63, 14195 Berlin, Germany; 4 Faculty of Health Sciences, Joint Faculty of the Brandenburg University of Technology Cottbus-Senftenberg, The Brandenburg Medical School Theodor Fontane, University of Potsdam, 14469 Potsdam, Germany

**Keywords:** cell-free protein synthesis, yeast, *Pichia pastoris*, protein production, energy regeneration, design of experiments, signal sequence, orthogonal system

## Abstract

Cell-free systems are particularly attractive for screening applications and the production of difficult-to-express proteins. However, the production of cell lysates is difficult to implement on a larger scale due to large time requirements, cultivation costs, and the supplementation of cell-free reactions with energy regeneration systems. Consequently, the methylotrophic yeast *Pichia pastoris*, which is widely used in recombinant protein production, was utilized in the present study to realize cell-free synthesis in a cost-effective manner. Sensitive disruption conditions were evaluated, and appropriate signal sequences for translocation into ER vesicles were identified. An alternative energy regeneration system based on fructose-1,6-bisphosphate was developed and a ~2-fold increase in protein production was observed. Using a statistical experiment design, the optimal composition of the cell-free reaction milieu was determined. Moreover, functional ion channels could be produced, and a G-protein-coupled receptor was site-specifically modified using the novel cell-free system. Finally, the established *P. pastoris* cell-free protein production system can economically produce complex proteins for biotechnological applications in a short time.

## 1. Introduction

*Pichia pastoris* has been established as a highly common organism for recombinant protein production. This yeast offers an ideal balance between genetic manipulability, streamlined protein production, and the ability to post-translationally modify proteins [1,2]. Straightforward manipulation of the yeast genome, compared to mammalian cells, allows for the introduction of foreign genes and metabolic engineering with reduced effort [3]. Another advantage is the availability of regulatable promoters, such as the methanol-inducible AOX1 promoter, as a highly controllable genetic switch that enables precise control of target protein expression [4]. *P. pastoris* is easily scalable and also known for its ability to produce high levels of recombinant proteins, efficiently secrete proteins, and realize complex post-translational modifications, including glycosylation [5,6,7]. The presence of yeast-like glycans can lead to adverse reactions when glycosylated proteins were applied for human applications [8]. In contrast to the model organism *Saccharomyces cerevisiae*, which produces proteins with high mannose glycosylation, *P. pastoris*-based protein production results in fewer immunogenic glycosylation patterns and can grow to higher cell densities [9]. The simple handling during cultivation and transformation, as well as a doubling time of 1–3 h, lead to time-saving protein production in *P. pastoris* [10].

**Figure 1:**
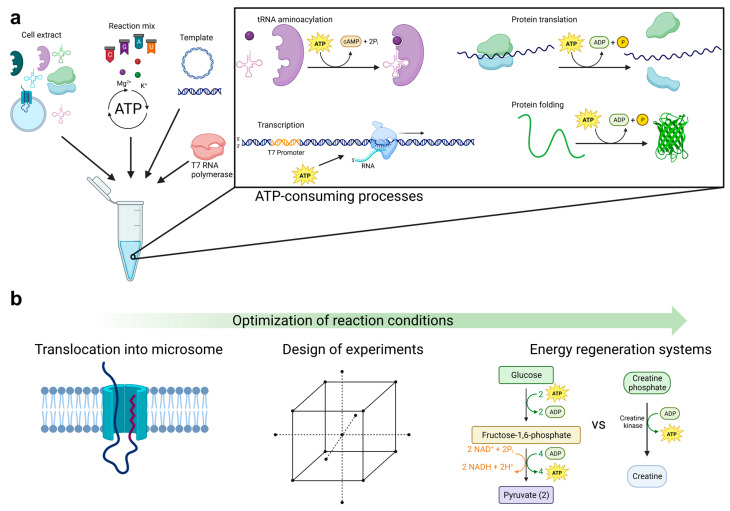
Cell-free protein synthesis with adjusted reaction conditions. (**a**) Eukaryotic cell-free protein synthesis is based on the utilization of a translationally active cell extract containing endogenous components, including aminoacyl tRNA synthetases, ribosomes, tRNAs, and microsomes. Moreover, a linear or circular DNA template, a T7 RNA polymerase, and a reaction mix containing defined concentrations of nucleotides, amino acids, and ions are needed. Additionally, an energy regeneration system is part of the reaction mix due to the ATP-consuming processes in cell-free protein synthesis. (**b**) Optimization strategies to obtain an effective *P. pastoris* cell-free protein synthesis system.

The properties of *P. pastoris* in recombinant protein production make it useful for cell-free synthesis of proteins, especially toxic proteins and membrane proteins [11,12]. Most importantly, it allows for the rapid production of proteins without depending on the growth and maintenance of living cell cultures. Proteins can be produced up to 60 µg/mL within 90–180 min in yeast-based cell-free systems, substantially accelerating the production of biotechnologically demanding proteins [13,14]. Furthermore, cell-free protein synthesis allows for precise control over production conditions, which can lead to higher yields and quality of the proteins produced [15]. In this approach, translationally active cell lysate, a reaction mix containing an energy regeneration system and substrates for transcription and translation, and T7 RNA polymerase are added to the open reaction environment to start cell-free protein synthesis based on a gene of interest downstream of a T7 promoter located on linear or circular DNA templates (Figure 1a). Other supplements can be added to the open system, such as detergents for solubilization of membrane proteins, the addition of components for site-specific modification of proteins, and cofactors to enhance protein activity [16].

Processes during cell-free protein production, including transcription, aminoacylation of tRNAs, ribosome dissociation, and protein folding, require ATP. Therefore, high-energy phosphor donors are often used to keep energy requirements in balance [17]. However, the reagents and energy sources required for cell-free protein synthesis are expensive, especially for large-scale protein production [18]. *Escherichia coli* represents a more economical way to produce proteins in cell-free reactions, also due to other energy regeneration systems. Although *E.coli* cell-free protein synthesis is widely used, the prokaryotic cell lysate offers limited post-translational modifications and thus fewer issues in the production of complex proteins [19]. On the other hand, post-translational modifications and optimal folding conditions are possible in eukaryotic cell-free protein production systems based on mammalian, insect, and tobacco cells [20,21,22]. Moreover, it was shown that membrane proteins can be translocated into vesicles derived from the endoplasmic reticulum (ER), termed microsomes, to facilitate the production of active ion channels in CHO and insect cell-free systems [23]. However, the cultivation and preparation of translationally active cell lysates is associated with increased time and cost, compared to *E. coli*-based cell-free protein synthesis. In fact, yeast-based cell-free protein synthesis can be used to produce complex proteins at a relatively low investment of both time and expense. In recent years, various protocols have been developed to establish cost-effective *S. cerevisiae*-based cell-free protein synthesis based on different cultivation, disruption, and lysate processing methods, as well as additives to the cell-free reaction [24,25,26]. Various protocols could be transferred to *P. pastoris*, and new methods could be established to use *P. pastoris* lysates in a cell-free format to synthesize, for instance, virus-like particles that are difficult to produce [27,28,29].

In the present work, *P. pastoris* cells were treated under different conditions to generate translationally active cell lysates. Evaluation of different signal sequences demonstrates the possibility of simultaneous production enhancement and translocation into microsomes (Figure 1b). A statistical experimental design was created to show that reaction conditions could be modified with a low time requirement and high informative value. An alternative source based on glycolysis intermediates for energy regeneration aimed to make the established cell-free yeast expression system more productive and less expensive to produce complex proteins, including the ion channels KvAP and KcSA.

## 2. Materials and Methods

### 2.1. Plasmids

The plasmids Adora2a-amb-Nluc and Adora2a-Nluc (with and without an amber stop codon at amino acid position 215) contain a melittin signal peptide and were used as previously described [30]. The plasmid DNA coding for the voltage-gated potassium channel KvAP and the potassium channel KcSA were used as previously described [31,32]. The ppα-GFP plasmid contains an α-mating factor prepro peptide fused to GFP and was purchased from Biocat. The firefly luciferase plasmid was utilized as previously described [33]. All plasmids for cell-free protein synthesis contain a cricket paralysis virus (CrPV) internal ribosome entry site (IRES) for translation initiation. The Mel-GFP plasmid containing a melittin signal peptide was utilized as previously described [34].

### 2.2. Yeast Cells and Cultivation

*P. pastoris* cells were obtained from the Leibniz Institute DSMZ—German Collection of Microorganisms and Cell Cultures GmbH (DSMZ no: 70382), while the *S. cerevisiae* strain S288C was obtained from the American Type Culture Collection (ATCC no: 204508). *P. pastoris* and *S. cerevisiae* cells were cultured at 29 °C and 300 rpm in YPD (1% yeast extract, 2% peptone, 2% dextrose) medium using baffled shake flasks, unless otherwise noted. Shake flasks were filled up to 20% culture volume capacity.

### 2.3. Cell Lysate Preparation

*P. pastoris* cells were harvested at a backscatter signal of ~5500 a.u. measured by the Cell Growth Quantifier (CGQ) online biomass monitoring system (Scientific Bioprocessing, Baesweiler, Germany). Cells were immediately cooled down to 4 °C and centrifuged for 10 min at 500× *g*. The cell pellet was washed three times with washing buffer (30 mM HEPES-KOH (pH 7.4), 100 mM KOAc, 2 mM DTT) using centrifugation steps of 5 min at 4 °C and 500× *g*. After the final wash step, for each 1 g wet cell pellet, 1 mL cell disruption buffer (30 mM HEPES-KOH (pH 7.4), 100 mM KOAc, 2 mM DTT), and 1 × cOmplete ULTRA Protease Inhibitor Cocktail (Roche, Basel, Switzerland) were used for resuspension. Bead-based homogenization was performed by adding 1 mL resuspended cells to a 2 mL lysing tube containing a lysing matrix (MP Biomedical, Irvine, CA, USA). The yeast cells were disrupted by applying four cycles of 35 s, unless otherwise noted, at 6 m/s using the FastPrep-24 Bead-Beating instrument (MP Biomedicals, Irvine, CA, USA) in the presence of dry ice in the cooling chamber. High-pressure homogenization was performed at 35 KPSI using the CF1 Cell Disruptor (Constant Systems, Daventry, United Kingdom) by applying one or two cycles at 4 °C. Disrupted cells were initially centrifuged at 1500× *g* at 4 °C for 5 min, followed by centrifugation of the supernatant for 10 min at 8000× *g* and 4 °C. The supernatant was isolated, and the cell lysates were supplemented with Baker’s yeast tRNA (final concentration of 5 μg/mL) and RNasin (Promega, Madison, WI, USA) Ribonuclease Inhibitor (final concentration of 150 U/mL). Cell lysates were stored at −80 °C. *S. cerevisiae* was disrupted by bead-based homogenization equivalent to *P. pastoris*.

### 2.4. Cell-Free Protein Synthesis

Initially performed cell-free reactions were composed of 35% *P. pastoris* cell lysate, 10 µM PolyG, 100 ng/µL plasmid, 30 mM HEPES-KOH (pH 7.4, Carl Roth, Karlsruhe, Germany), 6 mM magnesium acetate (Merck, Darmstadt, Germany), 150 mM potassium acetate (Merck, Darmstadt, Germany), 100 µM amino acids (Merck, Darmstadt, Germany), 250 µM spermidine (Roche, Basel, Switzerland), 2.5 mM Dithiothreitol (Life technologies, Darmstadt, Germany), 100 µg/mL creatine phosphokinase (Roche, Basel, Switzerland), 20 mM creatine phosphate (Roche, Basel, Switzerland), 0.5 mM ATP (Roche, Basel, Switzerland), 0.5 mM GTP (Roche, Basel, Switzerland), 0.5 mM of UTP (Roche, Basel, Switzerland), 0.5 mM CTP (Roche, Basel, Switzerland), and 1 U/μL T7 RNA polymerase (Agilent, Santa Clara, CA, USA). A total of 30 µM radioactive ^14^C-leucine (Perkin Elmer, Rodgau, Germany) was added to the reaction to enable the qualitative analysis of radio-labeled proteins by gel electrophoresis. Additionally, ^14^C isoleucine and ^14^C valine were added when indicated. Cell-free reactions were incubated for three hours at 28 °C and 600 rpm, unless otherwise noted. Cell-free reactions without the addition of plasmid to analyze translational activity of cell lysates using endogenous mRNA were modified by omitting the plasmid and T7 RNA polymerase. Orthogonal cell-free reactions were further supplemented with 2 µM mutant *E. coli* tyrosyl-tRNA synthetase [35,36] (eAzFRS), 3 µM orthogonal tRNAtyr, and 2 mM p-azido-L-phenylalanine (AzF). The purified eAzFRS and the orthogonal tRNAtyr were prepared as previously described in detail [30]. Creatine phosphokinase (Roche, Basel, Switzerland) and creatine phosphate (Roche, Basel, Switzerland) were substituted by either 20 mM glucose (Sigma-Aldrich, St. Louis, MI, USA), glucose-6-phosphate (Sigma-Aldrich, St. Louis, MI, USA), fructose-6-phosphate (Sigma-Aldrich, St. Louis, MI, USA), fructose-1,6-bisphosphate (Sigma-Aldrich, St. Louis, MI, USA), 3-phosphoglyceric acid (Sigma-Aldrich, St. Louis, MI, USA), phosphoenolpyruvate (Sigma-Aldrich, St. Louis, MI, USA), glycerol (Sigma-Aldrich, St. Louis, MI, USA), or glycerol-3-phosphate (Sigma-Aldrich, St. Louis, MI, USA) in cell-free reactions based on alternative energy regeneration systems. A total of 330 µM nicotinamide adenine dinucleotide (Sigma-Aldrich, St. Louis, MI, USA), 150 µM cyclic AMP (Sigma-Aldrich, St. Louis, MI, USA), and 20 mM potassium phosphate (Merck, Darmstadt, Germany) were added to cell-free reactions based on glycolytic intermediates, unless otherwise noted. Cell-free reactions with ppα-GFP were performed in 96-well plates, and fluorescence was measured using the LightCycler 96 instrument (Roche, Basel, Switzerland). Modifications of the cell-free reaction composition were mentioned in the text.

### 2.5. Design of Experiments and Data Analysis

The Minitab software version 21.4.1 was utilized to design and analyze the conducted experiments. The fluorescence signal produced from ppα-GFP was the analyzed response. A full factorial experimental design with three factors on two levels (presence or absence of the tested factor) was performed with three replicates. A total of 24 totals runs, including 8 base runs, were carried out in one block. A significance level of 0.05 was used to interpret the results. The central composite design was performed as a two-level factorial (half fraction) plan using 90 randomized total runs in three blocks and a rotatable design (distance of each axial point from the center (α = 2.82843)). The design included 64 cube points, 8 center points in the cube, 14 axial points, and 4 center points in the axis. Analysis of variance and backward elimination of terms was performed by using an α-to-remove value of 0.1 to generate a robust model.

### 2.6. Autoradiography of Radiolabeled Proteins

After cell-free protein synthesis, a 5 µL translation mixture was mixed with 45 µL of water, and proteins were precipitated by the addition of 150 µL acetone. Protein precipitation was performed on ice for 15 min, and the precipitated pellet was isolated by centrifugation for 10 min at 4 °C and 16,000× *g*. A total of 20 µL LDS sample buffer (Invitrogen, Carlsbad, CA, USA) containing 50 mM DTT was used to resuspended pellets after drying for 45 min at 45 °C to remove acetone. Samples were mixed at 1400 rpm for 30 min at room temperature. Denaturing polyacrylamide (14%) gel electrophoresis (PAGE) was based on the SureCast Gel Handcast System (Invitrogen, Carlsbad CA, USA). Subsequently gels were stained with SimplyBlue SafeStain (Thermo Scientific, Waltham, MA, USA) and dried by a Unigeldryer 3545D vacuum chamber (UniEquip, Martinsried, Germany) and deposited on phosphor screens (GE Healthcare, Chicago, IL, USA) for three days, and incubated screens were scanned by the Amersham Typhoon RGB (GE Healthcare, Chicago, IL, USA). The presence of ^14^C-labeled amino acids in cell-free reactions allowed for the visualization of the produced proteins based on autoradiography.

### 2.7. Fluorescence Microscopy

The translation mixture was centrifuged at 16,000× *g*, 4 °C, for 15 min to enrich the microsomes in the pellet. The supernatant was discarded, and the pellet was resuspended in an equal amount of PBS. The samples were transferred to an 18-well flat μ-Ibidi-Slide (Ibidi, Gräfelfing, Germany). The GFP fluorescence was visualized via excitation by a 488 nm laser, and emitted light was captured at 505 nm using an LSM 510 meta laser-scanning microscope (Zeiss, Oberkochen, Germany) equipped with a Plan-Aprochromat 63×/1.4 oil objective.

### 2.8. Luciferase Assay

Firefly luciferase activity was detected using Luciferase Assay Reagent (Promega, Madison, WI, USA). Therefore, 50 μL of reagent was added to 5 μL of translation mixture after cell-free protein synthesis, and luminescence was detected by the LB 941 luminometer (Berthold Technologies, Bad Wildbad, Germany). The concentration of active luciferase was determined using a calibration curve.

### 2.9. Electrophysiological Measurements

The translation mixture of cell-free-produced KcSA and KvAP was centrifuged for 10 min at 16,000× *g* at 4 °C and the pellet was resuspended in an equal-volume PBS. The resuspended pellet contained the microsomal vesicles and was utilized for electrophysiological measurements. Planar bilayer experiments were performed, as explained previously [37]. Lipid bilayers were formed from 1, 2-diphytanoyl-sn-glycero-3-phosphocholine (DPhPC) (Avanti Polar Lipids, Albaster, AL, USA). Lipids were dissolved in octane (Sigma Aldrich, Munich, Germany) at a concentration of 10 mg/mL. A total of 10 mM HEPES, pH 7.45, and 150 mM KCl (Sigma Aldrich (Fluka), Munich, Germany) were used as an electrolyte buffer. For KcSA recordings, a buffer with pH 4.0 was used. A total of 5 µL of the vesicles resuspended in PBS was added to the chamber containing the buffer, and we waited until there was a visible response. For current measurements, different voltages were applied to analyze the functional properties. The cavity contains the non-polarizable working electrode containing an Ag/AgCl layer deposited on the underlying Cr/Au layer. Briefly, 180 µL of electrolyte solution was added to the measurement chamber of an Orbit 16 system (Nanion Technologies, Munich, Germany). A single-channel amplifier (EPC-10, HEKA Electronic Dr. Schulze GmbH, Lambrecht, Germany) was connected to the multiplexer electronics port of the Orbit16 system. Recordings were performed at a sampling rate of 50 kHz with a 10 kHz Bessel filter. Data were analyzed with Clampfit (Molecular Devices, Sunnyvale, CA, USA).

## 3. Results

### 3.1. Enhancement of Protein Production with Cell-Free Pichia pastoris Systems by Specific Adjustment of Cell Disruption Conditions

**Figure 2:**
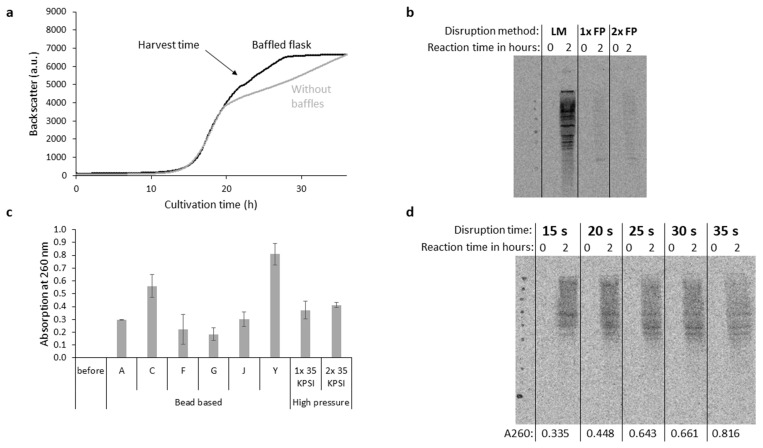
Identification of mild cell disruption parameters for cell lysate preparation. (**a**) Cell growth of *P. pastoris* cells was monitored over time by using an online biomass sensor. Cultivation in shake flasks with and without baffles was compared. The arrow indicates the harvest time when the yeast cells were disrupted for cell lysate preparation. (**b**) *P. pastoris* was disrupted by bead-based homogenization (lysing matrix C, LM) and high-pressure homogenization, referred to as French press (FP). Cell lysate was used for cell-free reactions containing initially ^14^C isoleucine, ^14^C leucine, and ^14^C valine to monitor protein translation of endogenous mRNA. After completion of the cell-free reaction the samples were precipitated by acetone, separated by SDS-PAGE, and visualized by autoradiography. (**c**) *P. pastoris* cells were disrupted with the indicated bead-based lysing matrix and French press. Cell disruption by using bead-based homogenization was carried out in four cycles of 35 s, while French press-based cell disruption was carried out at 35 KPSI in either one or two cycles. The absorption of the pre-diluted resulting cell lysate was measured at 260 nm. Measurements were performed in two independent cell lysate preparations. Data are shown as mean ± SD. (**d**) Bead-based homogenization was carried out in two cycles by using lysing matrix Y by varying the time for each of the cycles. Afterwards, the absorption was measured at 260 nm, and cell-free reactions containing ^14^C leucine were utilized to monitor protein translation of endogenous mRNA. Afterwards, samples were precipitated by acetone, separated by SDS-PAGE, and visualized by autoradiography.

Primarily, the growth behavior of the *P. pastoris* cells was examined. Therefore, online biomass monitoring was utilized to analyze cell growth over time in flasks with and without baffles (Figure 2a). As expected, growth in baffled flask resulted in an extended log phase due to increased aeriation. Although the highest growth rate was achieved after 16 h (Supplementary Figure S1), the cultivation was continued to 24 h and yeast cells were harvested at a backscatter signal of ~5500 a.u. to reach higher cell densities. In the next step, the aim was to identify a disruption method that could be used for processing a large number of yeast cells. While it has already been shown that high-pressure homogenization is effective in breaking the stable cell wall of *P. pastoris* while maintaining a translationally active lysate [38], bead-based homogenization with different bead compositions was also applied to gently disrupt the yeast cells. Bead-based homogenization was initially performed in a 2 mL lysing tube, but it can be scaled up to 50 mL tubes. The evaluation was performed via direct detection of the translational activity of the resulting cell lysates. For this purpose, the production of radioactively labeled proteins in the presence of the endogenous mRNA of the crude lysates by supplementation of ^14^C-labeled amino acids in cell-free protein synthesis was analyzed. After two hours of cell-free protein synthesis, the reaction was precipitated by acetone and compared against a reaction at 0 h based on an autoradiographic analysis of the running pattern in SDS-PAGE. In fact, the high-pressure homogenization showed only a low translational activity in contrast to the bead-based homogenization (Figure 2b). Therefore, further lysing matrix tubes with different materials were utilized to disrupt the yeast cells. The success of disruption was observed by the release of RNA, which resulted in increased absorption at 260 nm. Recognizable by the highest absorption signal, the disruption with 0.5 mm-diameter yttria-stabilized zirconium oxide beads (lysing matrix Y) was the most successful when applying identical amount of cycles (4×) and a disruption time of 35 s (Figure 2c). Subsequently, the disruption time during a cycle was varied, and only two cycles were applied to reduce the burden on the active components in the lysate, but we still obtained a high proportion of disrupted cells. Although a higher release of RNA was observed with increasing disruption time, the translational activity was almost equal, as indicated by the intensity of ^14^C leucine-labeled endogenous proteins in the autoradiograph (Figure 2d). Consequently, two cycles and a time of 15 s were used for the efficient disruption of *P. pastoris* cells in the following experiments. 

### 3.2. Effect of Signal Sequences on Cell-Free P. pastoris Synthesis

**Figure 3:**
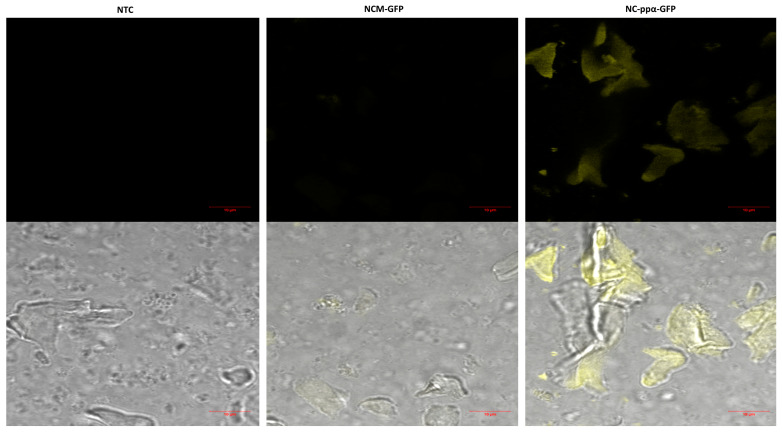
Visualization of protein translocation into ER derived microsomes. Cell-free reactions based on *P. pastoris* lysate were carried out in the presence of either no template (no template control, NTC), melittin signal peptide fused to GFP (Mel-GFP), or α-mating factor prepro peptide fused to GFP (ppα-GFP). Microsomes were centrifuged after the cell-free reaction and solubilized in PBS. Fluorescence was visualized via confocal microscopy after excitation at 488 nm. The excitation with the 488 nm laser is shown at the top, while an overlay of the bright field and the fluorescence image is shown at the bottom.

Signal sequences play an essential role in cell-free protein synthesis by allowing for the precise control of protein expression as well as the proper folding of proteins produced in the optimal environment. These signal peptides, also often referred to as translocation sequences, can direct the efficient translocation of the co- or post-translationally produced protein into the ER. The presence of microsomes in the processed *P. pastoris* lysate was detected by the presence of the ER marker NADPH cytochrome c reductase (Supplementary Figure S2). Honeybee melittin signal peptide (Mel) can be used to enhance the co-translational translocation of complex proteins in cell-free systems [39]. In contrast, in recombinant protein production using *P. pastoris* cells, the *S. cerevisiae* α-mating factor prepro peptide (ppα) is frequently adopted as a post-translational signal sequence [40,41,42]. In this study, the translocation of GFP with either of the two signal sequences was examined microscopically. A much higher fluorescence intensity of microsomal structures was detected by the N-terminal fusion of the ppα signal sequence in contrast to the weaker fluorescence signal for Mel-GFP (Figure 3). Nevertheless, an increase in the overall translational efficiency could be achieved with both signal sequences (Supplementary Figure S3).

### 3.3. Boosting the Performance of Yeast-Based Cell-Free Protein Synthesis through Alternative Energy Regeneration Systems

**Figure 4:**
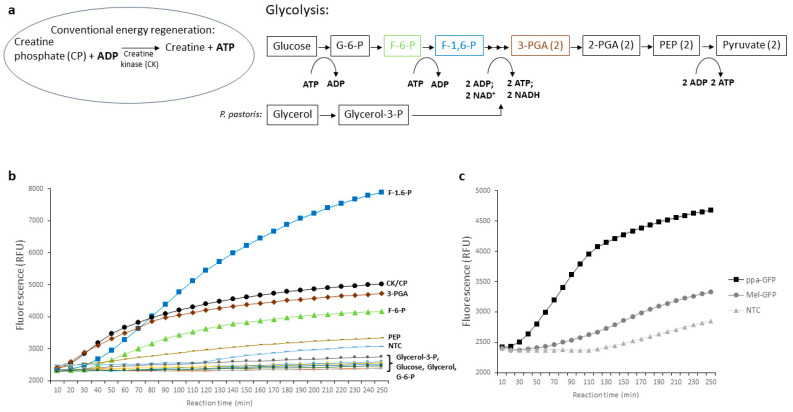
Alternative energy system for cell-free protein synthesis. (**a**) Scheme of the conventional energy regeneration in eukaryotic cell-free protein synthesis and an overview of glycolytic substrates for an alternative energy regeneration in *P. pastoris* cell-free systems. The colors of F-6-P (green), F-1,6-P (blue), and 3-PGA (brown) correspond to the graphs in (**b**). (**b**) Real-time fluorescence measurement of *P. pastoris* cell-free reactions containing ppα-GFP as template. The utilized energy source is indicated at each graph and is visualized in different colors to increase the readability. (**c**) Real-time fluorescence measurement of cell-free reactions based on *S. cerevisiae* lysate using F-1,6-P as energy source. Utilized templates were indicated as squares (ppα-GFP), circles (Mel-GFP), and triangles (no template control, NTC).

Maintaining an adequate energy level is essential for cell-free protein synthesis, as protein production requires energy-intensive processes. To meet this need, various approaches of energy regeneration have been used. A common method in eukaryotic cell-free protein synthesis is the use of substrates such as phosphoenolpyruvate (PEP), creatine phosphate (CP), and acetyl phosphate, which are enzymatically converted by the presence of the enzymes in the lysate or by enzyme addition [17]. These substrates provide an important source of high-energy compounds needed for protein synthesis. However, these substrates can pose a problem as high-energy phosphate bond donors due to their high cost and inhibitory effects on cell-free protein synthesis, which is mainly caused by the rapid production of phosphate [43,44]. Although it has been shown that *E. coli*-based cell-free synthesis can proceed efficiently with alternative energy sources, energy regeneration based on creatine kinase (CK) and CP is widely used in eukaryotic systems [45]. Alternative attempts to switch to inexpensive glycolysis intermediates have already been demonstrated with *S. cerevisiae* cell-free protein synthesis [46]. Anderson et al. were able to fuel their glucose energy system in the presence of supplemented cyclic AMP (cAMP) and exogenous phosphate. Additionally, nicotinamide adenine dinucleotide (NAD) is an important cofactor of glycolysis and is frequently supplemented in PANOx-based *E.coli* cell-free protein synthesis [47,48]. Therefore, NAD, cAMP, and potassium phosphate (KPO_4_) were added to cell-free reactions containing glycolytic intermediates. To examine whether energy from glycolysis can be harnessed for cell-free protein synthesis, the following glycolytic intermediates were tested: glucose, glucose-6-phosphate (G-6-P), fructose-6-phosphate (F-6-P), fructose-1,6-bisphosphate (F-1,6-P), 3-phosphoglyceric acid (3-PGA), and PEP. As glycerol is a carbon source commonly used in recombinant protein production for *P. pastoris*, the potential to enable cell-free protein synthesis via energy production from glycerol and glycerol-3-phosphate (Glycerol-3-P) was investigated (Figure 4a). Initial experiments with glycolysis intermediates in the *P. pastoris* cell-free reaction have shown that the use of the CP/CK system still works most efficiently (Supplementary Figure S4). Hence, the processing of the crude lysate was subsequently modified. Similar to *E. coli*-based cell-free production as reported previously [49,50], the dialysis and size exclusion chromatography were omitted in order to retain a large set of cell lysate components that may have a significant effect on glycolysis present in the cell-free reaction. The cell-free reaction was monitored in real time by using ppα-GFP as a template. Strikingly, GFP production was increased ~2-fold in the presence of F-1,6-P in contrast to the previously used CP/CK system (Figure 4b). Although it was shown that increased F-6-P and 3-PGA resulted in higher fluorescence signals, the supplemental addition of F-6-P and 3-PGA did not notably increase protein yields when 20 mM F-1,6-P was present in the cell-free reaction (Supplementary Figure S5). To demonstrate the broad application of F-1,6-P to other cell-free systems, identical parameters were transferred to a cell-free synthesis of GFP based on *S. cerevisiae* lysate. Indeed, GFP could be successfully produced in the presence of F-1,6-P (Figure 4c). Thus, transferability to other fungal-based cell-free systems is feasible. In the conventional energy regeneration system, CK and CP have to be supplemented, whereas in the novel system, the addition of F-1,6-P is sufficient through the presence of glycolytically active enzymes in the cell lysate.

### 3.4. Increasing the Efficiency of Cell-Free P. pastoris Protein Synthesis Using Statistical Experimental Designs

**Figure 5:**
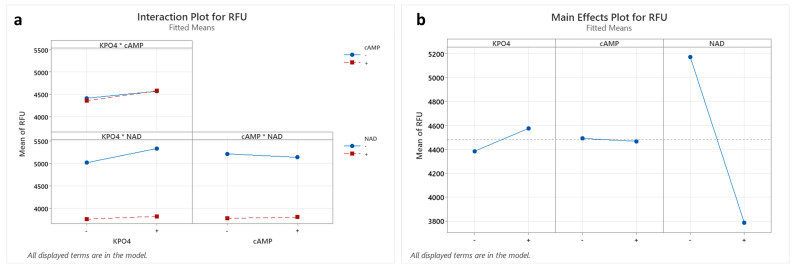
Full-factorial design evaluating the influence of NAD, cAMP, and KPO_4_ on cell-free protein synthesis. Cell-free reactions were carried out to evaluate the influence of NAD, cAMP, and KPO_4_ on general protein synthesis expressed through GFP fluorescence measurement. Therefore, a full-factorial design with two levels—absence (−) or presence (+) of the factor—was performed with three independent replicates. (**a**) Interaction plot. (**b**) Main effect plot.

Changing single parameters, such as glycolysis intermediates, buffer, and ions, to adjust the cell-free environment by the one-factor-at-a-time method is often time-consuming and costly but can also miss interactions between various parameters of cell-free protein synthesis. Consequently, there are numerous strategies to adjust the optimal composition for cell-free reactions based on statistical experimental designs. In addition, the use of crude lysate for cell-free synthesis raises the question of the optimal level of essential components. As a first step, we investigated whether the energy-regenerating system based on F-1,6-P can be influenced by the cofactors NAD, cAMP, and KPO_4_, similar to those reported previously [46]. A full factorial experimental design with two levels (presence or absence of the tested factor) was performed with three replicates. Significant two-way and three-way interactions could not be detected (Figure 5a and Supplementary Table S1). A significant negative effect on the overall synthesis performance was observed at higher NAD concentrations, while KPO_4_ and cAMP had no significant effect on cell-free protein synthesis at all (Figure 5b). Consequently, the three factors were not supplemented to the cell-free reaction for further experiments.

**Figure 6:**
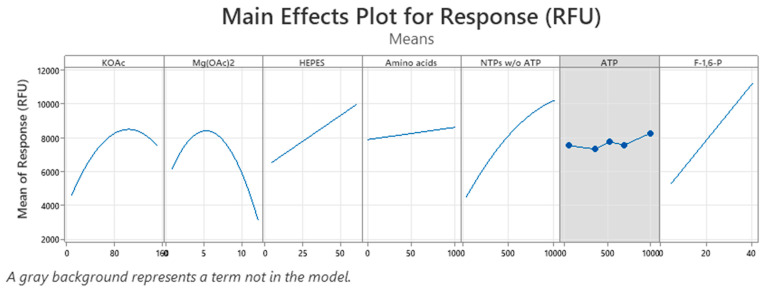
Main effect plots of the central composite design. Cell-free reactions were carried out in a 96-well plate, and fluorescence was measured as response to evaluate the central composite design based on seven factors. Main effect plots were generated for each factor. A white background represents a term which is in the generated model, while a grey background indicates a term which is not in the model. The x-axis indicates the tested concentration range of each factor.

Due to the large number of parameters affecting the cell-free reaction itself, we focused on factors which were shown to be essential in cell-free protein synthesis. Hence, HEPES (pH 7.4), potassium acetate (KOAc), magnesium acetate (Mg(OAc)_2_), adenosine triphosphate (ATP), nucleoside triphosphates without ATP (NTPs w/o ATP), F-1,6-P, and an amino acid mix were included in a central composite design to obtain meaningful information on the optimal composition of the cell-free reaction, while other components of the cell-free reaction were kept constant. A total of 90 cell-free reactions were carried out in a 96-well plate in the presence of a GFP template to evaluate the fluorescence signal as a response for the statistical analysis. A backward elimination with an α of 0.1 for removing terms from the model was utilized to create a robust model. The model explains 89.68% of the variation in the response and has a high predictive ability (R^2^-pred: 83.24%) for new observations (Supplementary Table S2). The model fits the experimental data, evidenced by the *p*-value 0.296 for the lack-of-fit test. The analysis of variance can be found in Table 1. Significant main effects (white background) are shown for all tested factors except for ATP, while the main effect of ATP was not significant (grey background) and was thus not included in the model (Figure 6). Moreover, a non-linear behavior, indicated by the curvature in the main effect plots, could be detected for KOAc, Mg(OAc)_2_, and NTPs w/o ATP. Significant interactions between KOAc and NTPs w/o ATP, as well between the energy regeneration system F-1,6-P and the factors Mg(OAc)_2_, HEPES, and NTPs w/o ATP, could be observed (Table 1 and Supplementary Figure S9). Further information can be found in Supplementary Figures S8–S10.

Based on the prediction of the generated model, we produced firefly luciferase in cell-free reactions to estimate protein yields using the established cell-free system. Therefore, *P. pastoris* cells were independently cultivated and disrupted. As a result, five different *P. pastoris* cell lysates were utilized to produce luciferase based on predicted optimal concentrations in *P. pastoris* cell-free reactions (Table 2). The mean protein concentration 24.5 ± 0.3 µg/mL of active luciferase could be determined. It is important to emphasize that no signal sequence was used, so the N-terminal fusion of Mel or ppα peptides could lead to higher protein yields.

### 3.5. Site-Specific Modification in P. pastoris Cell-Free Reactions by Using an Orthogonal Translation System

**Figure 7:**
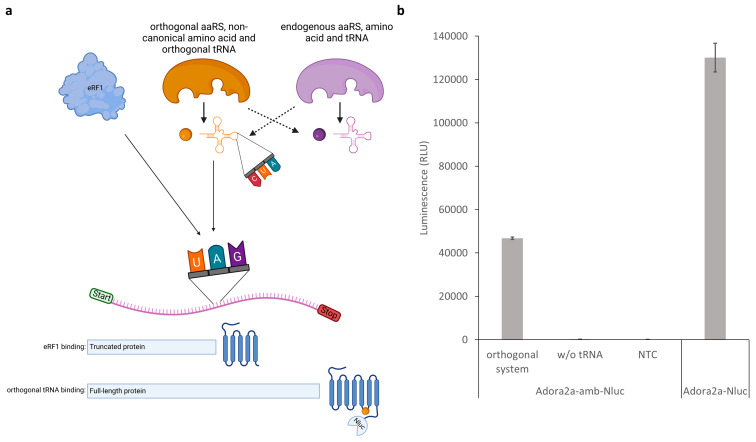
Site-specific protein modification in *P. pastoris* cell-free systems by using an orthogonal system. (**a**) Scheme of site-specific incorporation of a non-canonical amino acid into the GPCR Adora2a-amb-Nluc. Dashed arrows indicate no interaction. (**b**) The mutant orthogonal *E. coli* tyrosyl-tRNA synthetase, orthogonal tRNAtyr, and p-azido-L-phenylalanine (AzF) were added to a cell-free reaction based on *P. pastoris* lysate containing the Adora2a-amb-Nluc template. A no-template control (NTC) and a control reaction without orthogonal tRNAtyr were utilized. Adora2a-Nluc without an amber stop codon was used to examine the amber suppression efficiency. Luminescence was detected after completed cell-free reactions. Measurements were performed in technical triplicate. Data are shown as mean ± SD.

To demonstrate the broader scope of the novel system, the possibility of modifying proteins at defined amino acid positions was investigated. Site-specific modification of proteins in cell-free protein synthesis is of high importance as it allows for the directed adaptation of proteins to specific functions. By introducing non-canonical amino acids at precisely defined sites in the protein, the activity, stability, or interaction with other molecules can be tailored [51,52]. This opens up possibilities for the development of customized biotechnological applications, such as therapeutic proteins with improved properties [53]. The orthogonal aminoacyl tRNA synthetase (aaRS), orthogonal tRNA, and the non-canonical amino acid (ncaa) necessary for site-specific modification can be conveniently added to the cell-free reaction without consideration of cell viability, as in cell-based applications [54,55]. In this process, the endogenous aaRS, tRNAs, and amino acids must not cross-react with the orthogonal components (Figure 7a). A competition for the recognition of the amber stop codon exists between aminoacylated orthogonal tRNA and the eukaryotic release factor (eRF1) to either suppress the amber stop codon or terminate protein translation. Since the present novel cell-free system based on *P. pastoris* is intended to be a cost-effective alternative for the production of complex proteins, we further aimed to demonstrate that the system is also suitable to modify complex proteins in a target-specific manner. For this purpose, the mutant orthogonal *E. coli* tyrosyl-tRNA synthetase/tRNAtyr pair was used, which is frequently employed in eukaryotic-cell-based and cell-free systems to modify proteins site-specifically with diverse ncaa such as p-azido-L-phenylalanine (AzF) and p-propargyloxyphenylalanine [30,35,36]. The pharmacologically relevant G-protein-coupled adenosine receptor A2a (Adora2a) was site-specifically modified in a cell-free *P. pastoris* synthesis by the insertion of an amber stop codon at amino acid position 215. C-terminal fusion of a nanoluciferase (Adora2a-amb-Nluc) was used to determine successful amber stop codon suppression by detecting luminescence. The suppression efficiency could be obtained by comparing the synthesis of Adora2a-amb-Nluc with a cell-free reaction of Adora2a with a C-terminal nanoluciferase without an amber stop codon in between (Adora2a-Nluc). While a suppression efficiency of 36% was achieved, no amber stop codon readthrough was observed in the absence of orthogonal tRNAtyr, indicating the high specificity of the orthogonal system (Figure 7b).

### 3.6. Functional Characterization of the Potassium Channels KcSA and KvAP Reconstituted into Planar Lipid Bilayers

Due to the presence of microsomes, we aimed to produce functionally active ion channels using the *P. pastoris* cell-free system. Planar lipid bilayer-based reconstitution is widely used for measuring the functionality of potassium channels [56,57]. Planar lipid bilayer electrophysiology involves creating a lipid bilayer across a cavity, dividing it into two compartments with buffer solutions on both sides. After forming the bilayer, the sample, including proteins like KcSA and KvAP, is added. This results in the reconstitution of the ion channels in the planar lipid bilayer. Subsequently, a voltage clamp is applied to initiate the gating of ion channels between open and closed states, facilitating the transport of ions. The movement of ions generates a current, which is recorded during measurements.

**Figure 8:**
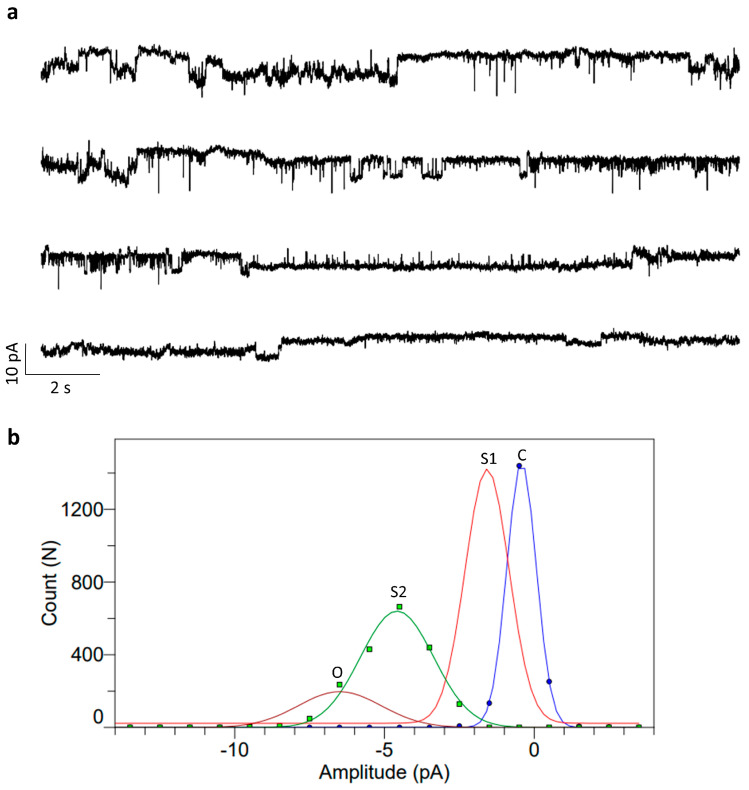
Planar lipid bilayer electrophysiology measurements from the DPhPC bilayers after the addition of KcSA containing vesicles. (**a**) Representative current traces of single-channel activity of KcsA in pure DPhPC bilayers at pH 4.0 (N > 5) at −100 mV. (**b**) All time amplitude histograms of traces of the above single-channel trace showing multi-conductance peaks (C: closed; S1: sub-conductance 1; S2: sub-conductance 2; and O: full open conductance). Buffer: 10 mM HEPES, 150 mM KCl, pH 4.0.

Following the addition of the KcSA-incorporated microsomal fraction to the DPhPC lipid bilayer, we observed transient, discrete conductance changes in the form of single-channel conductance with amplitudes of 5–7 pA at −100 mV (Figure 8a). Electrophysiological recordings showed a clear single channel activity with several sub-conductance levels at different voltages, similar to McGuire and Blunck [57]. In between, there are several sub-conductance levels (2–3 pA) rapidly flickering between the closed and half levels of the full-conductance state. All-time histograms were plotted for all recordings, and we noticed sub-conductance to full-conductance peaks corresponding to the KcSA channel activity. Similar types of currents recorded at +60 mV are also shown in Supplementary Figure S11, with a histogram showing two peaks corresponding to the opening and closing of the channel. We were able to observe a range of conductance behaviors ranging from a large peak corresponding to closed levels and several smaller peaks corresponding to sub-conductance, single-channel, and cooperative multi-conductance gating levels (Figure 8b). Multi-conductance levels might correspond to the insertion of more than a single channel. Moreover, several macro currents were observed, which were not shown in this draft as they are often irrelevant and most often end up in the rupturing of the underlying lipid bilayers. These macro currents might either be due to the rapid insertion of several ion channels or to the osmotic stress of the microsomal fraction. Rarely, these macro currents were also seen in negative controls, where only a sample without the expressed protein was added.

**Figure 9:**
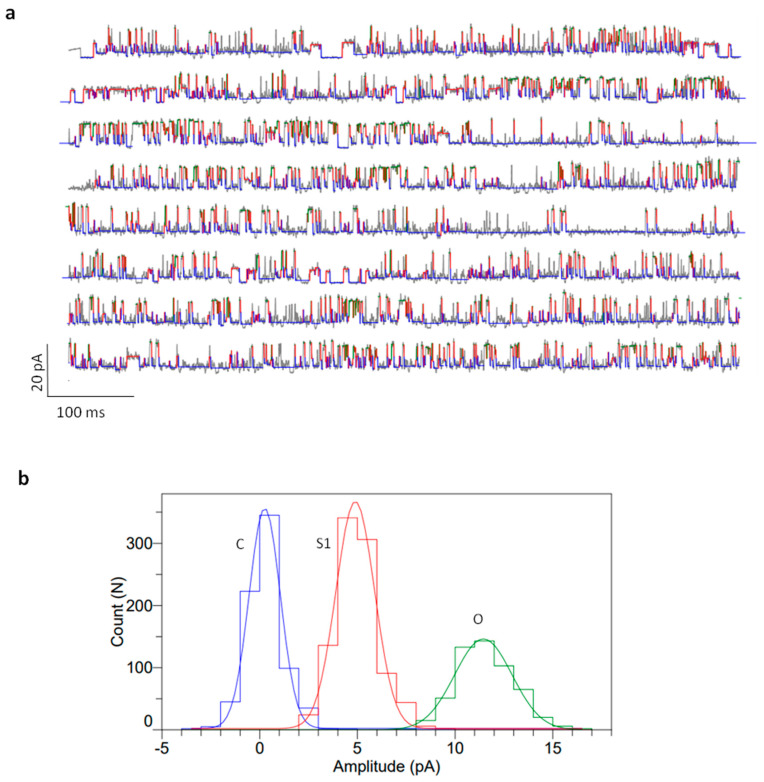
Planar lipid bilayer electrophysiology measurements from the DPhPC bilayers after the addition of KvAP-containing vesicles. (**a**) Representative current traces of single-channel activity of KvAP in pure DPhPC bilayers at pH 7.45 (N > 5) at +100 mV. The color corresponds to the conductance peaks in (**b**). (**b**) All time amplitude histograms of traces of the above single-channel trace showing multi-conductance peaks (C: closed in blue; S1: sub-conductance 1 in red; and O: full open conductance in green). Buffer: 10 mM HEPES, 150 mM KCl, pH 7.45.

KvAP activity was measured after adding the microsomal fraction harboring the active protein onto the planar lipid bilayer. After several minutes, there were different types of response observed. We focused on measuring the single-channel recordings from the reconstituted lipid bilayers. Single-channel conductance was measured from the KvAP-reconstituted DPhPC lipid bilayers, as shown in Figure 9a. The measured currents correspond to a single channel conductance of around 120 pS, with several sub-conductance levels in between, transitioning from full-length conductance to closed conductance in accordance with Devaraneni et al. [58]. All time histograms showed multi-conductance peaks corresponding to the partially opened channel (5 pA) and full-length conductance (12 pA) (Figure 9b). Similar activity was also observed in a different experiment at +60 mV (Supplementary Figure S12a,b). In the controls, there were some leaky large currents that increased over time, resulting in the rupturing of the bilayer. These events were also observed with the same frequency in the case of KvAP-reconstituted lipid bilayers; hence, they were considered unrelated to the presence of KvAP. However, in the case of KvAP samples, macroscopic currents resulting from the stochastic opening of several channels were observed, showing rectification of the currents in one polarity. These macroscopic currents varied from bilayer to bilayer and were not considered for analysis.

## 4. Discussion

*P. pastoris* plays a prominent role in recombinant protein production. Although the scalability of eukaryotic cell-free systems for the production of difficult-to-produce proteins is limited due to high costs, a more economical low-cost system would be of outstanding interest in the manufacture of proteins on a larger scale. In the present work, the developed cell-free protein production system based on the yeast *P. pastoris* could provide a low-cost alternative to already established eukaryotic cell-free systems.

In the present study, we demonstrated that the N-terminal fusion of Mel and ppα signal sequences can achieve increased protein production and translocation into microsomes, allowing diverse complex proteins to be optimally folded and post-translationally modified. In this process, the pre sequence is expected to be removed by signal peptidases in the ER, while the pro sequence is cleaved in the Golgi. However, it is reported that ppα-signaling peptides can lead to accumulation outside of the ER [59]. On the other hand, different mutant ppα-signaling peptides were established [60] and could conceivably be used in the future for cell-free synthesis, providing efficient translocation during recombinant protein production.

With the use of the novel energy regeneration system, which utilizes F-1,6-P as a substrate, cell-free protein synthesis can be realized even more economically. On the one hand, F-1,6-P is also a high-energy phosphate bond donor, but in contrast to the widely used substrate CP, there is no need for additional CK supplementation since a large proportion of enzymes of glycolysis seem to be present in the cell lysate in an active form. The fact that the cell lysate can be directly used for cell-free protein synthesis without further chromatographic purification means that a comparably low time requirement as for *E. coli* cell lysate generation is necessary. Interestingly, the use of F-1,6-P could be transferred to *S. cerevisiae* cell-free protein synthesis without further optimization, empowering cell-free protein synthesis based on this model organism. Hence, it is conceivable to substitute the CK/CP system by F-1,6-P for other cell-free systems.

In fact, it was shown in this study that supplementation of cAMP and KPO_4_, as well as NAD as an important cofactor of glycolysis, had no effect or a negative effect, respectively, on the production of GFP, although a positive effect on *S. cerevisiae*-based cell-free synthesis has already been shown [46]. Therefore, it can be assumed that the components are present in sufficient and balanced form in the cell lysate, which are otherwise unavailable for the cell-free reaction due to dialysis and chromatography. These results were in accordance with a previous report on *E. coli*-based cell-free reactions using F-1,6-P as an energy source without NAD and coenzyme A addition [61].

The capability of running cell-free syntheses in a 96-well format has shown that 90 reactions can be performed in parallel to enable statistical analyses of the optimal environment of the reaction components. This means that the cell-free system can be adapted to new conditions with little effort and at low cost. The model constructed here represents a high predictive power, with an R^2^-pred of 83.24%, and the predicted optimal concentrations of HEPES, Mg(OAc)_2_, ATP, NTPs w/o ATP, and amino acids are in agreement with the recent results of a statistical experiment with cell-free protein synthesis based on *P. pastoris* [62]. It is noticeable that increasing HEPES and NTPs without ATP and F-1,6-P levels resulted in elevated protein synthesis. The preference for higher HEPES concentration may be caused by the omitted rebuffering of the lysate, whereas the optimum at high NTP concentrations could be explained by a high lysate concentration, as described by Takahashi et al. [63]. In addition, the high F-1,6-P concentration seems to function optimally as an energy source for ATP. Among other things, magnesium ions are important cofactors in complexes with ATP for glycolytic enzymes such as aldolase, phosphoglycerate kinase, and pyruvate kinase, explaining the significant interactions of F-1-,6-P with Mg(OAc)_2_ [64]. Surprisingly, no significant interaction of ATP with magnesium ions was detected, although it is known that high ATP levels can affect magnesium-dependent processes, such as protein synthesis, considerably owing to their high affinity for magnesium ions [65,66]. This could indicate that higher ATP concentrations than assayed might have a negative effect on protein synthesis.

Although cell-free *P. pastoris* protein synthesis functions through the presence of endogenous amino acids, the addition of exogenous amino acids was shown to have a positive effect on GFP production. Therefore, substitution with a more economical amino acid source would be desirable. Recently, it has been shown that cell-free syntheses based on *E. coli*, *Streptomyces venezuelae*, and *P. pastoris* by common nutrient-rich media such as peptone can be effectively used as an amino acid source for cell-free protein production [67].

Site-specific modification allows for precise control of chemical changes at defined sites, facilitating the production of tailored proteins with improved properties and functions, for instance, to produce antibody-drug conjugates [68]. Previously it was shown that AzF was incorporated into trastuzumab IgG to establish a yeast-based platform for the production of antibody-drug conjugates [69]. While it has already been shown that cell-free orthogonal translation systems have the potential to modify difficult-to-produce proteins [30,70], the present results demonstrate that the cell-free site-specific modification of difficult-to-produce proteins in *P. pastoris* lysates is associated with significantly lower costs.

Given that *P. pastoris* cells are susceptible to genetic modification, cell-free synthesis can be improved by inserting diverse beneficial protein-coding sequences into the genome. It was shown that T7 RNA polymerase was inserted into the *P. pastoris* genome under the control of a constitutively expressing GAP promoter to drive transcription via a T7 promoter in yeast cells [71]. Additionally we recently showed that inserting the orthogonal aaRS into the CHO genome can reduce costs and time, while achieving sufficient amber suppression to site-specifically modify membrane proteins [72]. Hence, incorporating these enzymes would strengthen the novel yeast cell-free protein synthesis.

The potential to use a cell-free *P. pastoris* system for recombinant protein production has already been demonstrated by Aw and Polizzi [38]. By overexpressing the global regulator of ribosome biogenesis FHL1, a 3-fold increase in protein yields could be achieved. Furthermore, using their developed cell-free *P. pastoris* system, Wang et al. recently demonstrated that CrPV IRES can initiate protein translation most efficiently among the 14 IRESs tested [29].

In summary, a cost-effective *P. pastoris* cell-free system was successfully established to produce functionally active complex proteins. Furthermore, it was also demonstrated that this system can be used for targeted protein modification, which opens up promising possibilities for future biotechnological applications.

## 5. Conclusions

In this study, we identified efficient cell disruption conditions to produce translationally active cell lysate for cell-free protein synthesis based on *P. pastoris*. The cell-free environment was optimized via the statistical experimental design, and the translocation of proteins into ER vesicles was shown. Furthermore, we utilized the mutant orthogonal *E. coli* tyrosyl-tRNA synthetase/tRNAtyr pair to introduce AzF into the GPCR Adora2a-amb-Nluc. In addition, we increased the protein yield by ~2-fold using fructose-1,6-bisphosphate as an energy regeneration system. Finally, the novel system was shown to produce active ion channels.

## Figures and Tables

**Table 1:** Model summary and analysis of variance of the central composite design.

Source	*p*-Value
Model	0.000
Linear	0.000
KOAc	0.000
Mg(OAc)_2_	0.000
HEPES	0.000
Amino acids	0.094
NTPs w/o ATP	0.000
F-1,6-P	0.000
Square	0.000
KOAc*KOAc	0.000
Mg(OAc)_2_*Mg(OAc)_2_	0.000
NTPs w/o ATP*NTPs w/o ATP	0.078
Two-Way Interaction	0.000
KOAc*NTPs w/o ATP	0.001
Mg(OAc)_2_*F-1,6-P	0.000
HEPES*F-1,6-P	0.003
NTPs w/o ATP*F-1,6-P	0.000
Error	
Lack-of-Fit	0.296
Pure Error	
Total	

**Table 2:** Predicted optimal concentrations for *P. pastoris* cell-free protein synthesis. Tested parameters are indicated in grey.

Components	Predicted Final Concentration
KOAc	141.5 mM
HEPES pH 7.4	60 mM
Mg(OAc)_2_	7.6 mM
F-1,6-P	40 mM
NTPs w/o ATP	1 mM
Amino acids	100 µM
ATP	0.5 mM
T7 RNA Polymerase	1 U/µL
*P. pastoris* lysate	35%
Spermidine	0.25 mM
PolyG	5 µM
DTT	1 mM
Nanoluciferase plasmid	100 ng/µL

## Data Availability

Data is contained within the article or supplementary material.

## Supplementary Materials

The following supporting information can be downloaded at https://www.mdpi.com/article/10.3390/bioengineering11010092/s1. Supplementary Figure S1: Growth rate of P. pastoris. P. pastoris was cultivated in shake flasks with (black dots) and without baffles (grey dots). Cell growth was monitored over time and growth rates were calculated using an online biomass sensor. Supplementary Figure S2: Cytochrome c Reductase (NADPH) assay. P. pastoris cell lysate was prepared and the Cytochrome c Reductase (NADPH) activity was determined using the Cytochrome c Reductase (NADPH) assay (Sigma-Aldrich) according to the manufacturer’s instruction. A positive control containing Cytochrome c Reductase (NADPH) from rabbit liver was additionally analyzed. Measurements were performed in technical duplicate. Data are shown as mean ± SD. Supplementary Figure S3: Cell-free protein synthesis with and without melittin signal peptide. EYFP and the ETB receptor were synthesized in P. pastoris cell-free reactions in the presence of 14C leucine. A melittin signal peptide (Mel) was inserted (+) or was omitted (−) in utilized templates. Proteins in the translation mixture were precipitated by acetone after cell-free protein synthesis. The autoradiography of radiolabelled proteins is visualized. Supplementary Figure S4: Cell-free protein synthesis based on different energy sources. Nanoluciferase (Promega) was synthesized in cell-free reactions based on P. pastoris cell lysate, which was processed by size exclusion chromatography. Luminescence was analyzed after cell-free protein synthesis by the Nano-Glo Luciferase Assay System (Promega). Measurements were performed in technical duplicate. Data are shown as mean ± SD. Supplementary Figure S5: Cell-free protein synthesis containing F-1,6-P. Cell-free synthesis of ppα-GFP based on P. pastoris cell lysate was performed in the presence of 20 mM F-1,6-P. Different concentrations of F-6-P and 3-PGA were applied during cell-free reactions. Fluorescence was detected after cell-free protein synthesis. Results are displayed as endpoint measurement. Supplementary Figure S6: Model equation of the full-factorial design. The model equation corresponds to Supplementary Table S1 and Figure S5 and is shown in uncoded units. Supplementary Figure S7: Residual plots of the full-factorial design. The influence of NAD, cAMP and KPO4 were analyzed based on a full-factorial design. Corresponding model information, analysis of variance and plots can be found in Supplementary Table S1 and Figure S5, respectively. Residual plots are shown and were prepared using Minitab software. Supplementary Figure S8: Model equation of the central composite design. The model equation corresponds to Table 1 and is shown in uncoded units. Supplementary Figure S9: Interaction plots of the central composite design. Interaction plots of the central composite design corresponding to Table 1 are shown and were prepared using Minitab software. Supplementary Figure S10: Residual plots of the central composite design. Residual plots corresponding to Table 1 are shown and were prepared using Minitab software. Supplementary Figure S11. Planar lipid bilayer electrophysiology measurements from the DPhPC bilayers after the addition of KcSA containing vesicles. (A) Representative current traces of single channel activity of KcsA in pure DPhPC bilayers at pH 4.0 (N > 5) at −100 mV. (B) All time amplitude histograms of traces of the above single channel trace showing multi-conductance peaks (C: closed, and C: full open conductance). Buffer: 10 mM HEPES, 150 mM KCl, pH 4.0. Supplementary Figure S12. Planar lipid bilayer electrophysiology measurements from the DPhPC bilayers after the addition of KvAP containing vesicles. (A) Representative current traces of single channel activity of KvAP in pure DPhPC bilayers at pH 7.45 (N > 5) at +60 mV. (B) All time amplitude histograms of traces of the above single channel trace showing multi-conductance peaks (C: closed, S1: sub-conductance 1 and C: full open conductance). Buffer: 10 mM HEPES, 150 mM KCl, pH 7.45. Supplementary Table S1: Model summary and analysis of variance of the full-factorial design. Supplementary Table S2: Model summary and analysis of variance of the central composite design corresponding to Table 1.
