# Supplementary material for: A Cost-Effective Pichia pastoris Cell-Free System Driven by Glycolytic Intermediates Enables the Production of Complex Eukaryotic Proteins
Source: Bioengineering (Basel). 2024 Jan 18;11(1):92. doi: 10.3390/bioengineering11010092 (PMC10813726; doi:10.3390/bioengineering11010092)
Supplement: Supplementary file 1 [file bioengineering-11-00092-s001.zip › bioengineering-2800991-supplementary.pdf]

Supplementary Materials

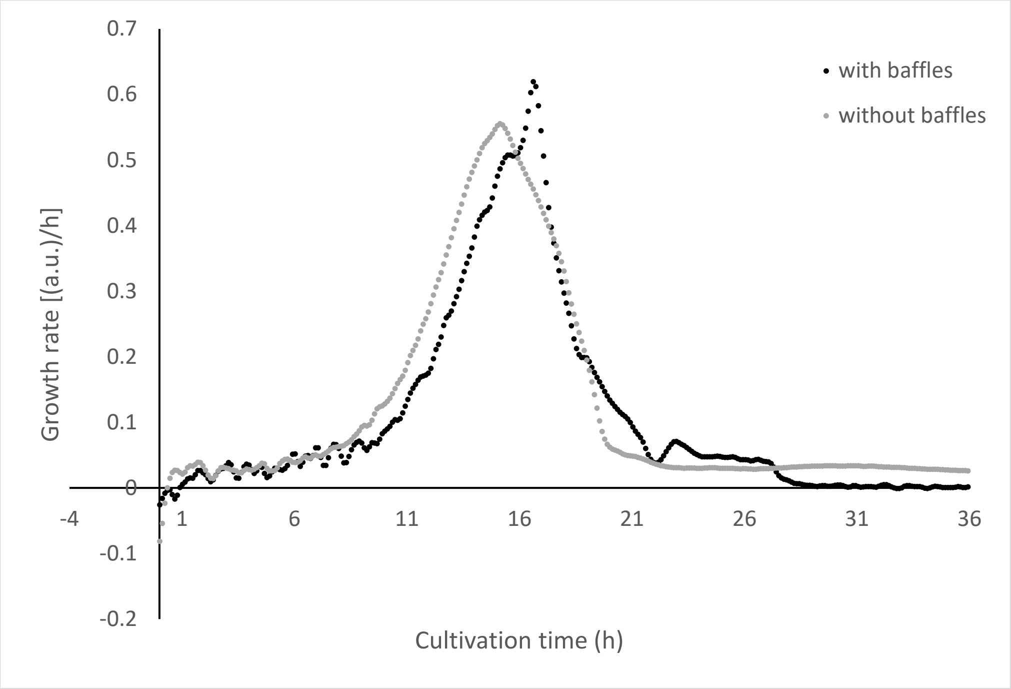

Supplementary Figure S1: Growth rate of P. pastoris. *P. pastoris* was cultivated in shake flasks with (black dots) and without baffles (grey dots). Cell growth was monitored over time and growth rates were calculated using an online biomass sensor.

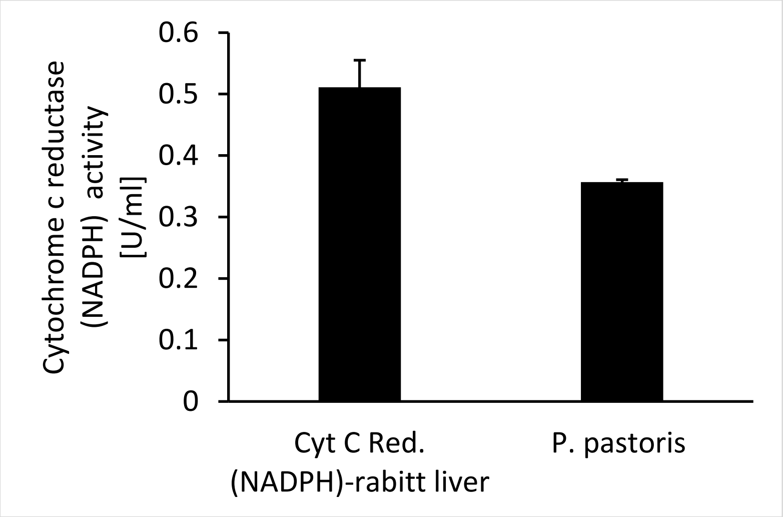

Supplementary Figure S2: Cytochrome c Reductase (NADPH) assay. *P. pastoris* cell lysate was prepared and the Cytochrome c Reductase (NADPH) activity was determined using the Cytochrome c Reductase (NADPH) assay (Sigma-Aldrich) according to the manufacturer's instruction. A positive control containing Cytochrome c Reductase (NADPH) from rabbit liver was additionally analyzed. Measurements were performed in technical duplicate. Data are shown as mean ± SD.

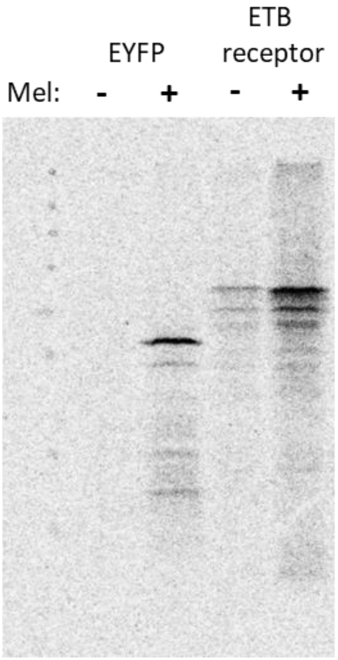

Supplementary Figure S3: Cell-free protein synthesis with and without melittin signal peptide. EYFP and the ETB receptor were synthesized in *P. pastoris* cell-free reactions in the presence of ^{14}C leucine. A melittin signal peptide (Mel) was inserted (+) or was omitted (-) in utilized templates. Proteins in the translation mixture were precipitated by acetone after cell-free protein synthesis. The autoradiography of radiolabelled proteins is visualized

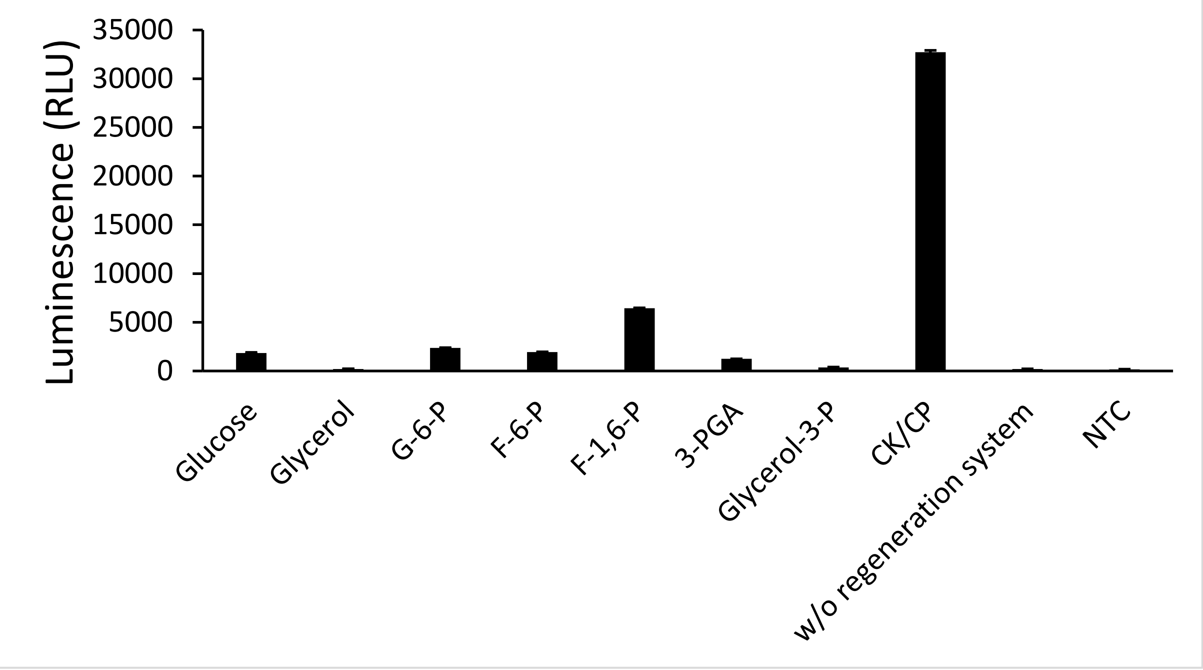

Supplementary Figure S4: Cell-free protein synthesis based on different energy sources. Nanoluciferase (Promega) was synthesized in cell-free reactions based on *P. pastoris* cell lysate, which was processed by size exclusion chromatography. Luminescence was analyzed

after cell-free protein synthesis by the Nano-Glo Luciferase Assay System (Promega). Measurements were performed in technical duplicate. Data are shown as mean ± SD.

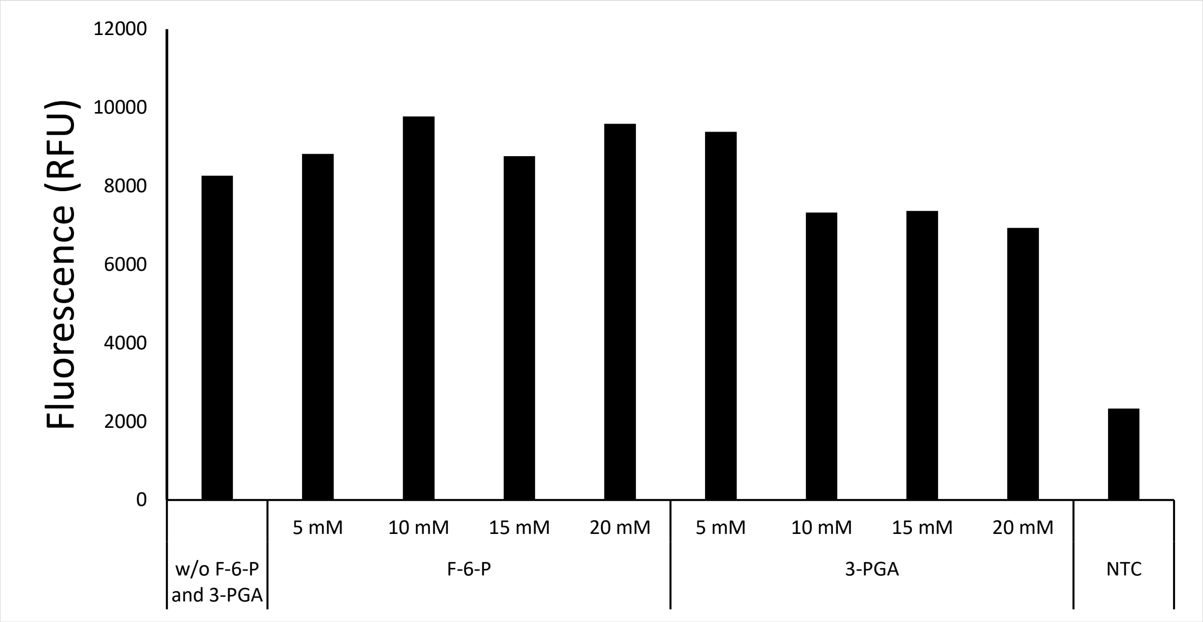

Supplementary Figure S5: Cell-free protein synthesis containing F-1,6-P. Cell-free synthesis of ppα-GFP based on *P. pastoris* cell lysate was performed in the presence of 20 mM F-1,6-P. Different concentrations of F-6-P and 3-PGA were applied during cell-free reactions. Fluorescence was detected after cell-free protein synthesis. Results are displayed as endpoint measurement.

Response (RFU) = 4480 + 95 KPO4 – 12 cAMP – 693 NAD + 17 KPO4*cAMP – 64 KPO4*NAD + 27 cAMP*NAD – 4 KPO4*cAMP*NAD

Supplementary Figure S6: Model equation of the full-factorial design. The model equation corresponds to Supplementary Table 1 and Figure 5 and is shown in uncoded units.

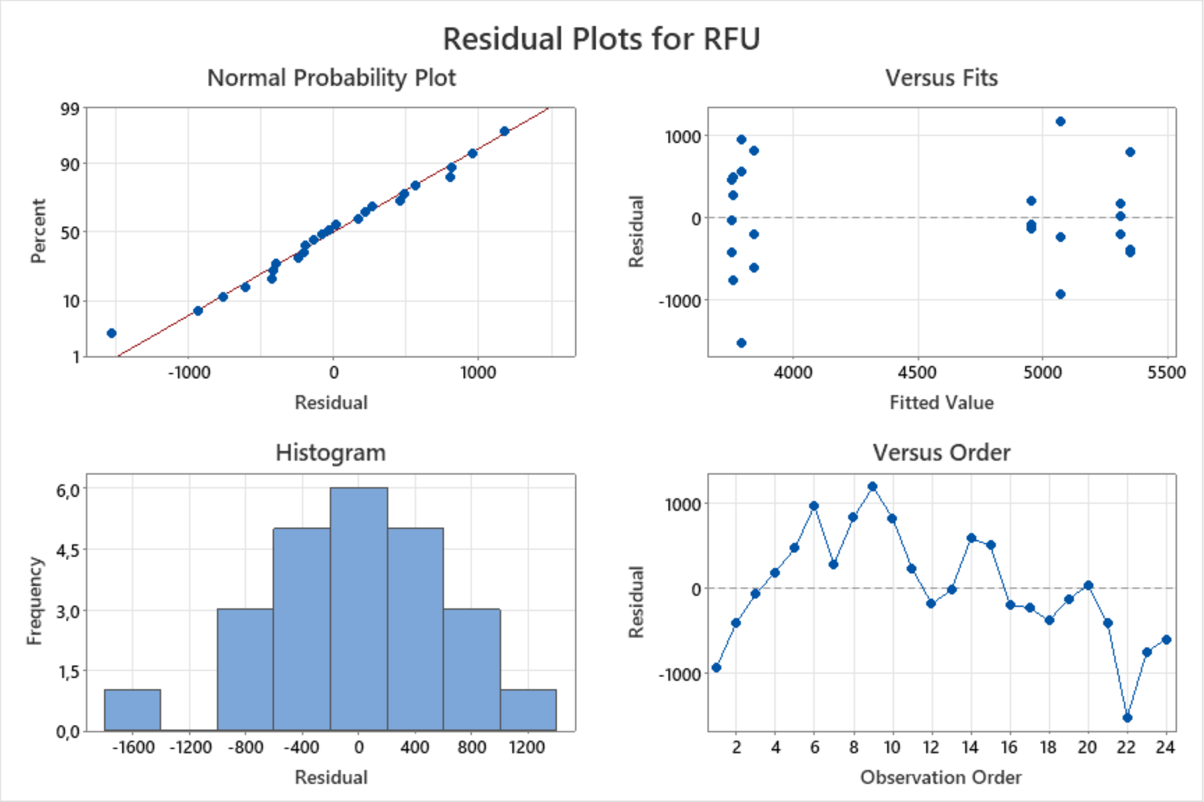

Supplementary Figure S7: Residual plots of the full-factorial design. The influence of NAD, cAMP and KPO_4 were analyzed based on a full-factorial design. Corresponding model information, analysis of variance and plots can be found in Supplementary Table 1 and Figure 5, respectively. Residual plots are shown and were prepared using Minitab software.

Response (RFU) = 5783 + 53.6 KOAc + 569 Mg(OAc)2 – 36.2 HEPES + 7.23 Amino acids – 5.01 NTPs w/o ATP – 386.2 F-1,6-P – 0.4263 KOAc*KOAc – 114.1 Mg(OAc)2*Mg(OAc)2 – 0.00386 NTPs w/o ATP*NTPs w/o ATP + 0.0667 KOAc*NTPs w/o ATP + 28.68 Mg(OAc)2*F-1,6-P + 4.34 HEPES*F-1,6-P + 0.4285 NTPs w/o ATP*F-1,6-P

Supplementary Figure S8: Model equation of the central composite design. The model equation corresponds to Table 1 and is shown in uncoded units.

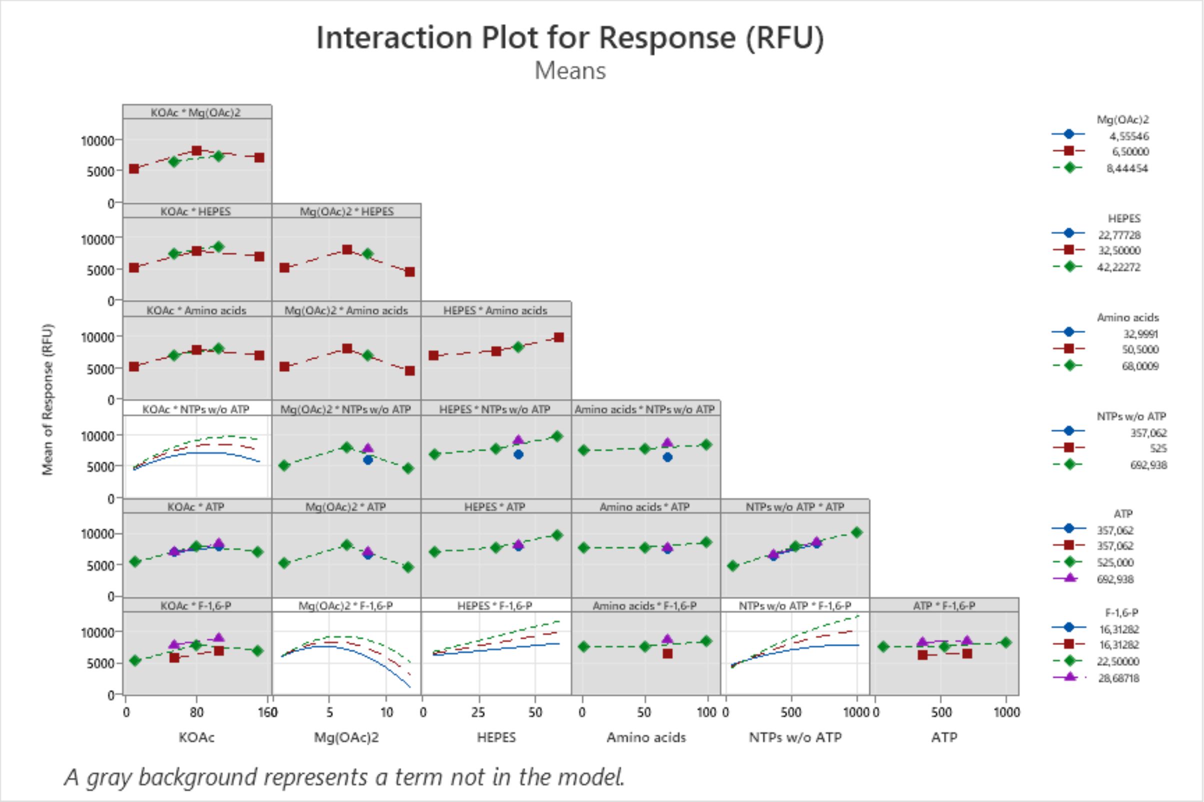

Supplementary Figure S9: Interaction plots of the central composite design. Interaction plots of the central composite design corresponding to Table 1 are shown and were prepared using Minitab software.

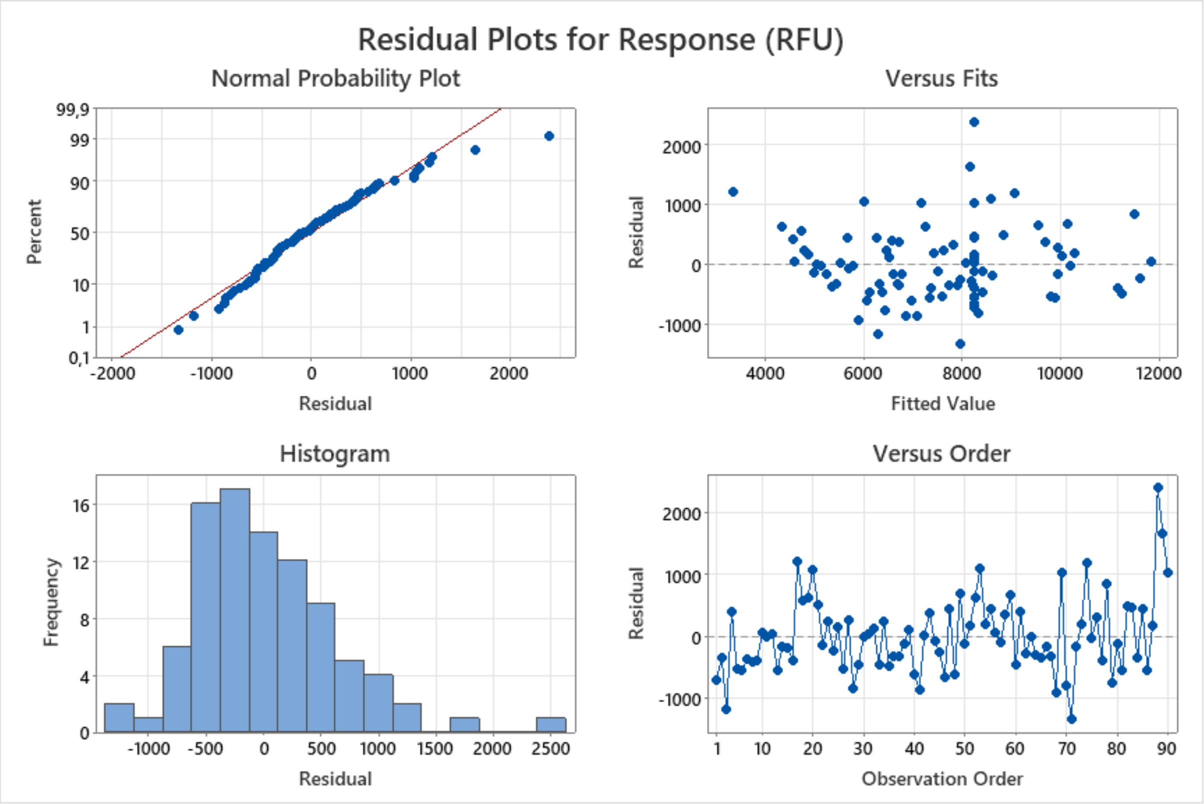

Supplementary Figure S10: Residual plots of the central composite design. Residual plots corresponding to Table 1 are shown and were prepared using Minitab software.

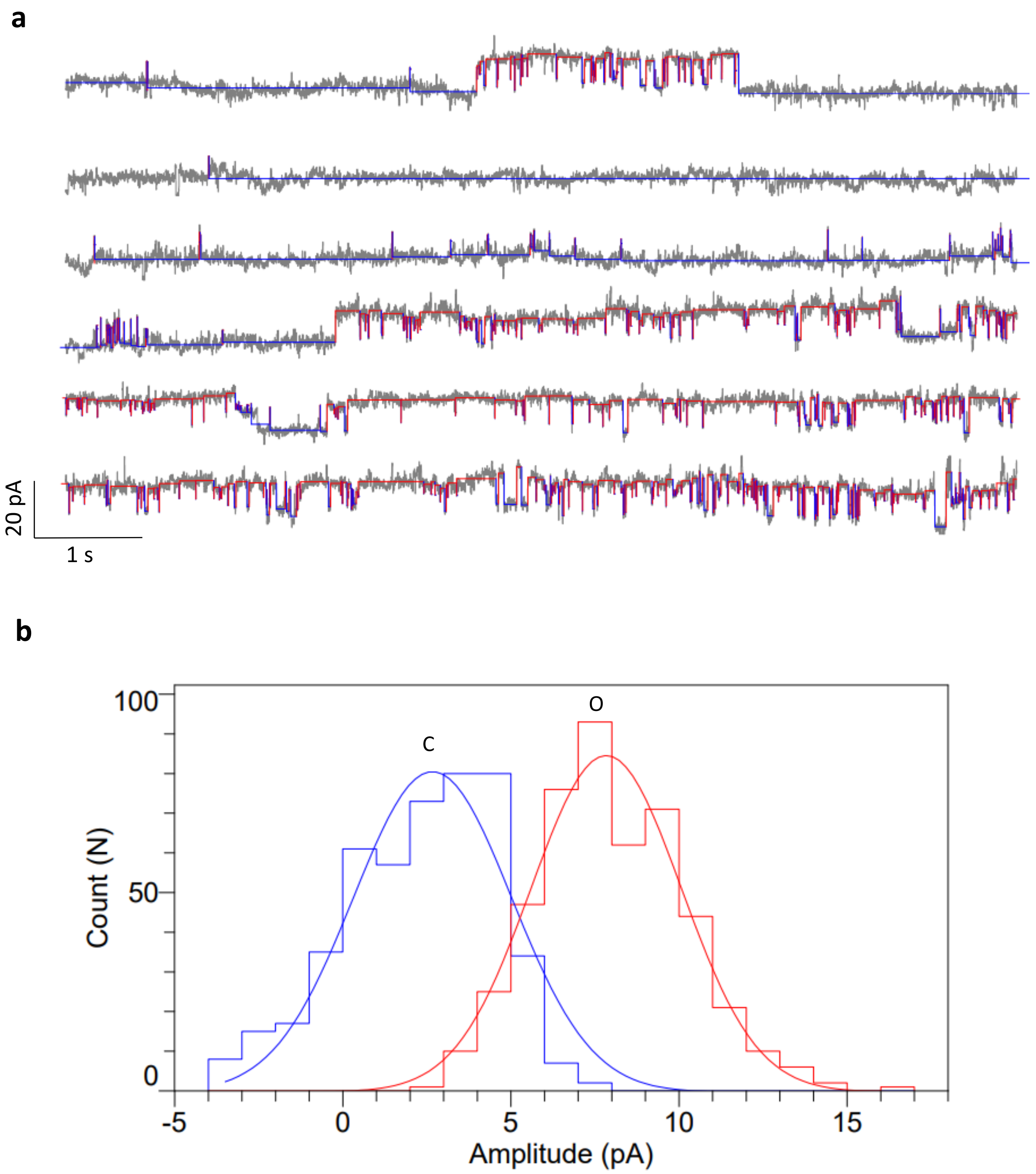

Supplementary Figure S11. Planar lipid bilayer electrophysiology measurements from the DPhPC bilayers after the addition of KcSA containing vesicles. A) Representative current traces of single channel activity of KcsA in pure DPhPC bilayers at pH 4.0 (N > 5) at -100 mV. B) All time amplitude histograms of traces of the above single channel trace showing multi-

conductance peaks (C: closed, and C : full open conductance). Buffer: 10mM HEPES, 150mM KCl, pH 4.0.

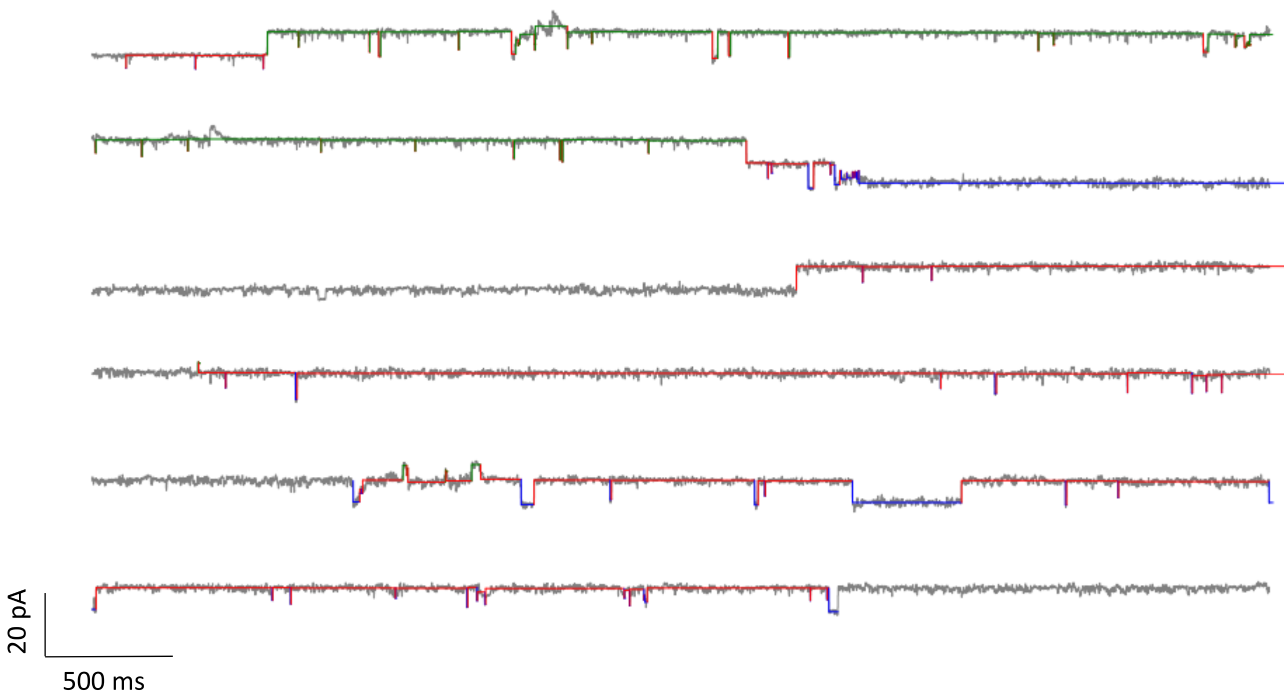

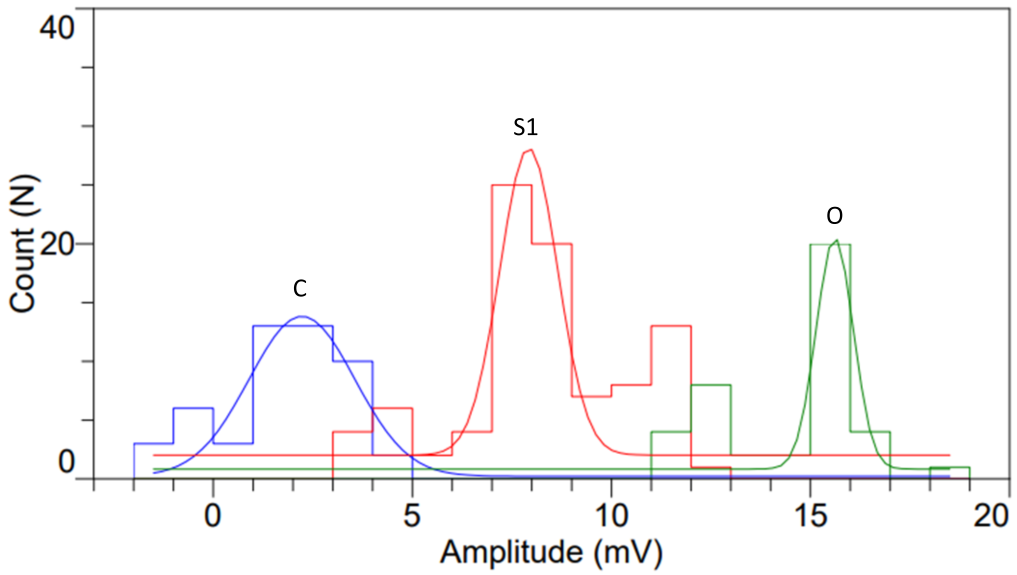

Supplementary Figure S12. Planar lipid bilayer electrophysiology measurements from the DPhPC bilayers after the addition of KvAP containing vesicles. A) Representative current traces of single channel activity of KvAP in pure DPhPC bilayers at pH 7.45 (N > 5) at +60 mV. B) All time amplitude histograms of traces of the above single channel trace showing multi-conductance peaks (C: closed, S1: sub-conductance 1 and C : full open conductance). Buffer: 10mM HEPES, 150mM KCl, pH 7.45.

Supplementary Table S1: Model summary and analysis of variance of the full-factorial design.

S	R^2	R^2-adj	R^2-pred
764.548	55.90%	36.61%	0.77%

Source	DF	Adj SS	Adj MS	F-Value	P-Value
Model	7	11854617	1693517	2,90	0,037
Linear	3	11733296	3911099	6,69	0,004
KPO4	1	217313	217313	0,37	0,551
cAMP	1	3433	3433	0,01	0,940
NAD	1	11512550	11512550	19,70	0,000
2-Way Interactions	3	120912	40304	0,07	0,976
KPO4*cAMP	1	6575	6575	0,01	0,917
KPO4*NAD	1	97080	97080	0,17	0,689
cAMP*NAD	1	17258	17258	0,03	0,866
3-Way Interactions	1	409	409	0,00	0,979
KPO4*cAMP*NAD	1	409	409	0,00	0,979
Error	16	9352537	584534		
Total	23	21207154			

Supplementary Table S2: Model summary and analysis of variance of the central composite design corresponding to table 1.

S	R^2	R^2-adj	PRESS	R^2-pred	AICc	BIC
667.62	89.68 %	87.92%	55025830	83.24%	1447.35	1478.36

Source	DF	Seq SS	Contribution	Adj SS	Adj MS	F-Value	P-Value
Model	13	294419365	89.68%	294419365	22647643	50.81	0.000
Linear	6	235352852	71.69%	235352852	39225475	88.00	0.000
KOAc	1	20355056	6.20%	20355056	20355056	45.67	0.000
$Mg(OAc)_2$	1	21780696	6.63%	21780696	21780696	48.87	0.000
HEPES	1	28466325	8.67%	28466325	28466325	63.87	0.000
Amino acids	1	1279414	0.39%	1279414	1279414	2.87	0.094
NTPs w/o ATP	1	78940714	24.05%	78940714	78940714	177.11	0.000
F-1,6-P	1	84530647	25.75%	84530647	84530647	189.65	0.000
Square	3	29484887	8.98%	29484887	9828296	22.05	0.000
KOAc*KOAc	1	6359531	1.94%	8182454	8182454	18.36	0.000
$Mg(OAc)_2$*$Mg(OAc)_2$	1	21706273	6.61%	22319426	22319426	50.07	0.000
NTPs w/o ATP*NTPs w/o ATP	1	1419083	0.43%	1419083	1419083	3.18	0.078
2-Way Interaction	4	29581625	9.01%	29581625	7395406	16.59	0.000
KOAc*NTPs w/o ATP	1	4914213	1.50%	4914213	4914213	11.03	0.001
$Mg(OAc)_2$*F-1,6-P	1	7620995	2.32%	7620995	7620995	17.10	0.000
HEPES*F-1,6-P	1	4356863	1.33%	4356863	4356863	9.77	0.003
NTPs w/o ATP*F-1,6-P	1	12689554	3.87%	12689554	12689554	28.47	0.000
Error	76	33874843	10.32%	33874843	445722		
Lack-of-Fit	67	30952666	9.43%	30952666	461980	1.42	0.296
Pure Error	9	2922177	0.89%	2922177	324686		
Total	89	328294207	100.00%				
